# Shedding light on functional near-infrared spectroscopy and open science practices

**DOI:** 10.1117/1.NPh.10.2.023520

**Published:** 2023-04-17

**Authors:** Caroline Kelsey, Jebediah Taylor, Laura Pirazzoli, Renata Di Lorenzo, Eileen F. Sullivan, Charles A. Nelson

**Affiliations:** a Boston Children’s Hospital, Department of Pediatrics, Division of Developmental Medicine, Boston, Massachusetts, United States; b Harvard Medical School, Boston, Massachusetts, United States; c Harvard Graduate School of Education, Cambridge, Massachusetts, United States

**Keywords:** functional near-infrared spectroscopy, neuroimaging, open science

## Abstract

Open science practices work to increase methodological rigor, transparency, and replicability of published findings. We aim to reflect on what the functional near-infrared spectroscopy (fNIRS) community has done to promote open science practices in fNIRS research and set goals to accomplish over the next 10 years.

Functional near-infrared spectroscopy (fNIRS) is an optical imaging technique that allows one to indirectly measure brain response through the assessment of oxygenated and deoxygenated hemoglobin concentration changes. fNIRS researchers are united in their goals to progress the understanding of the brain and share this knowledge widely, and one way to further the mission is through open science practices.

The open science movement is working to promote practices that strengthen methodological rigor, increase transparency in methods and analysis, disseminate null results, and ultimately, improve the replicability of published findings. Open science practices are now widely being adopted by many fields of research from basic biology to clinical and translational research.^1^^–^^3^ This review aims to reflect on what the fNIRS community has done so far to promote open science practices in fNIRS research and set goals to accomplish over the next 10 years.

To do this, this review will go through the research life cycle of an fNIRS research project from study inception to publication, highlighting ways in which fNIRS researchers have been implementing open science research practices and ways in which the field could be doing more. This review also aims to acknowledge potential challenges and concerns that laboratories face when adopting open science practices. The suggestions in this report are not meant to be condemning of others’ current research practices in any way and are by no means a “cure-all” for all problems faced by fNIRS researchers. Rather, the hope is to spark reflection and discussion about open science practices within labs and across the fNIRS field.

## 1 Preregistration

Preregistration is a means of creating documentation of the research plan before data collection or analysis. Preregistering studies increases transparency about the initial hypotheses and analysis plans (versus those developed *post hoc*) and constrains researcher degrees of freedom.^4^^,^^5^ Preregistration documentation can be submitted to a publicly available repository or registry. (These are a few registries available: arXiv, Dryad, Figshare, GitHub, Mendeley, Neuroimaging Tools and Resources Collaboratory, open science framework, rOpenSci). Currently, multiple user-friendly online platforms, such as the Open Science Framework (OSF),^6^ provide simple workflows for creating timestamped preregistrations. The typical preregistration plan contains the following information: hypotheses, target sample size and power analysis, data collection procedures and measures, planned analyses, and inference criteria (for an overview and guide to the practice see Ref. 7). The preregistration process has several options that allow flexibility in use. These include options to write up a secondary data preregistration for projects where the data are already collected but the researchers have yet to process or analyze the data; as well as the possibility to make the preregistration public immediately or after an embargo period, allowing researchers to release the information when they are ready. This process has many benefits to fNIRS researchers including (a) helping with study conceptualization and providing a time for reflection as to why fNIRS is the appropriate tool for a given question or how best to make use of fNIRS data, (b) limiting the need (or temptation) to run multiple processing streams to clean the data (e.g., by selecting specific methods and thresholds for channel pruning, motion correction, and artifact removal), (c) reducing the need (or temptation) to conduct multiple analyses (by choosing a particular analytic approach, e.g., cluster analysis, multivariate pattern analysis, functional channel of interest), (d) focusing analyses on specific regions of interest defined *a priori* and decreasing overall channel search space, (e) improving organization and communication for collaborative, multiresearcher endeavors, and (f) aiding in the ability to publish null or unexpected results (e.g., an inverted hemodynamic response) and increasing the trust and reliability of the findings.^8^^,^^9^ Before submitting a preregistration, it may be beneficial to run an initial pilot study or to run parameters through a simulated dataset. This can help to inform criteria selection and avoid potential missteps, including selecting criteria that are too strict (e.g., infants must look at 100% of the trials to be included, no channels are included after preprocessing, etc.), which could lead to insufficient sample size or a realization that the data are not well-suited for the analysis plan (e.g., non-normal distribution, not enough trials due to overly stringent inclusion parameters). Here, it is important to emphasize that preregistration is not meant to hamper scientific progress. If hypotheses change during the research cycle due to new evidence being published or new methodologies being developed, one is not restricted from adding new measures. Rather, researchers are encouraged to be transparent and explicit about what was preregistered versus what was exploratory (or added *post hoc*).

### 1.1 How Widely Adopted Is This Practice in the fNIRS Community?

**Fig. 1:**
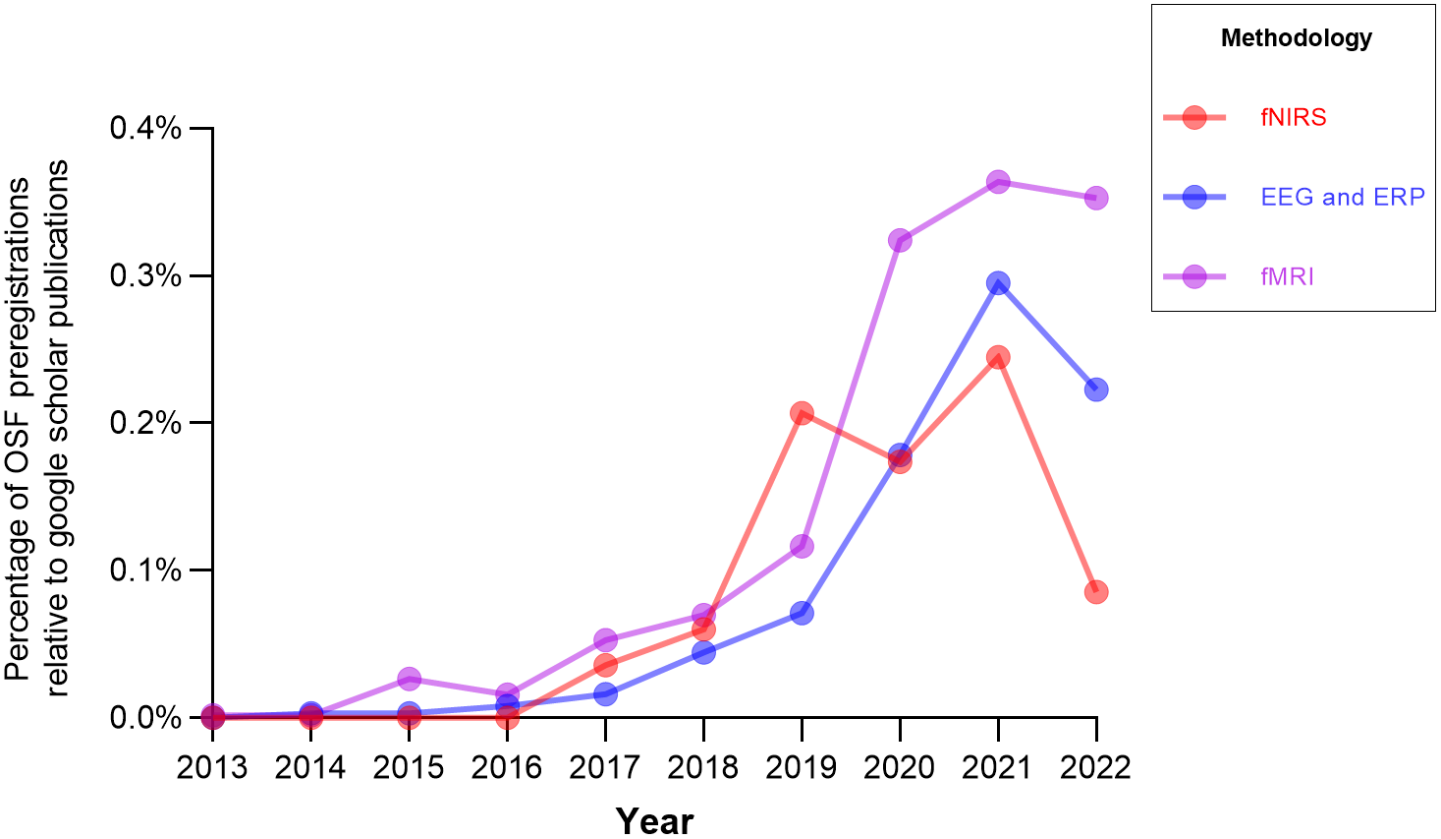
The ratio of preregistrations to publications for each imaging modality by year. Note: This search was conducted on March 29, 2022, using Ref. 10 (restricted to OSF Registries) and Google Scholar. This only includes preregistrations that have been made publicly available. The following search terms were used: fNIRS *or* “functional near-infrared spectroscopy,” fMRI *or* “functional magnetic resonance imaging,” EEG *or* electroencephalogram *or* ERP *or* “event related potential.”

Overall, there were significantly fewer preregistrations listed on the OSF registry written about fNIRS (see Table S1 in the Supplemental Material) compared with other widely used neuroimaging methodologies, electroencephalography (EEG), and functional magnetic resonance imaging (fMRI). However, when accounting for the total number of Google Scholar publications (used as an approximation for users across these modalities; see Fig. 1) as part of a weighted average, there are relatively similar rates of this practice. In addition, there is an upward trend such that preregistration is becoming generally more common across all three imaging modalities. Notably, the majority of fNIRS preregistrations followed a pre-existing template (82.7%) and the majority of preregistrations were done prior to data collection (2.5% were listed as a secondary data registration).

### 1.2 Potential Challenges and Considerations

It is important to acknowledge that preregistration involves extra work and planning and creates a new burden on the researcher. To recognize and reward these efforts, incentive programs could be put into place.

### 1.3 Goals for the Future

Looking ahead, the field could benefit from having a standardized fNIRS-specific preregistration template and an incentives program. Templates for fMRI and EEG have already been developed, and there have been recent efforts to develop an fNIRS preregistration template.^11^^–^^13^ The fNIRS-specific preregistration could have a structured form field that asks about specific fNIRS protocols (see Table 1 for examples). The template could help to inform researchers of the information that they need to include and provide standardization across labs. Having the template could also provide clarity on specific decision points. For example, there is a lot of variation in which chromophore, oxygenated or deoxygenated hemoglobin, is reported across studies. Therefore, the template could be of help by providing a standard language for hypotheses (e.g., an effect will be present in at least one chromophore) and recommendations for analyses (e.g., all analyses should include both chromophores). The fNIRS-specific template would also benefit from following the general guidelines for fNIRS research recommended by Ref. 14.

**Table 1:** Examples of information that could be included in an fNIRS preregistration.

Form fields	Examples of the types of information to include
**Study setup**	
fNIRS hardware	The fNIRS system used, the source–detector layout relative to the 10–20 system, and sizes of caps available
fNIRS software	Data acquisition software and presentation software
Study design details	Is this a block, event-related, or resting state study?
Paradigm details	The stimuli set, length of stimuli, and number of trials
Metadata that will be collected	Will head circumference measurements, hair color, or head shape be documented?
Auxiliary measurements	Will short channel or accelerometer data be recorded?
Cap placement	How are caps placed on a participant’s head (e.g., in reference to fiducial markers)? How is cap placement recorded (e.g., pictures, 3D scanners, video recordings, etc.)?
Lab environment	Information regarding the lab setup [e.g., the size of the screen, the lights in the room, who is in the room during the recordings, and objects in the room (e.g., toys for an infant to hold)]
Incidentals that could impact the hemodynamic signal	Documentation about specific contingency plans (e.g., infants are allowed to breastfeed if they are fussy, or experimenters will blow bubbles)
**Data processing**	
Behavioral coding	The types of behaviors which will be excluded from the time frame (e.g., experimental interference)
Channel exclusion	The channel exclusion parameters (e.g., detection of heart rate signal, etc.)
Motion detection correction and rejection	The parameters for motion detection and approach to motion correction [spline, wavelet, targeted principal component analysis (TPCA), prewhitening, etc.]
Modeling function for the hemodynamic response	GLM or block average? Will auxiliary regressors be used?
Chromophore(s) of interest	Note, it is suggested that all studies report effects for both oxygenated and deoxygenated hemoglobin
Exclusion criteria for a trial	The maximum amount of motion and the minimum amount of looking time needed to be included
Exclusion criteria for a participant	The number of usable trials that are needed per condition
**Data analysis**	
Regions of interest	A description of probe layout and which channels will be used
Statistical analysis methods	The types of statistical methods used (e.g., functional channel of interest, multivariate pattern analysis, functional connectivity network computation, general linear mixed models, etc.)

In addition, the fNIRS field could benefit from creating an incentives program (e.g., a badge program, see Ref. 15 for more information). Badges are a symbolic recognition given to the researchers, and they serve as a signal of values and beliefs held by the particular publisher.^16^ Essentially, for each open science practice a paper follows, (e.g., preregistration, sharing materials) they receive an additional badge. This easy-to-implement and no-cost badge program has been shown to be effective in increasing the number of papers that endorse open science practices.^17^ In addition, the fNIRS community could create fNIRS-specific badges to reward practices unique to the methodology (e.g., the analysis pipeline was preregistered and made publicly available). Preregistration could also be incentivized through incorporation into the peer-review process by having it as part of the checklist for reviewers and something that can be considered when evaluating a manuscript.

## 2 Registered Reports

Registered reports are a type of manuscript where the first round of peer review occurs after the study is designed but before data are collected or analyzed (referred to as a stage 1 manuscript). Therefore, peer review at this stage focuses on the theoretical framework and quality of methods rather than the outcome of the study, which can reduce publication bias and certain questionable research practices.^18^ It is at this stage that the editorial team may decide to accept (or reject) the paper, on the condition that the authors follow through with the registered methodology. After data collection and analysis, the research team resubmits the full paper (referred to as a stage 2 manuscript) and reviewers are asked to evaluate the manuscript on adherence to the protocol and whether interpretations are supported by the data. Similar to preregistrations, many journals also allow unplanned analyses to be included in the stage 2 manuscript as long as their exploratory nature is clearly stated. The registered report manuscript format has many benefits to fNIRS researchers including receiving feedback at an early stage where changes to probe layout, paradigm design, data collection, and data analysis can still be implemented. This early feedback is of considerable importance given the rapid progress in all aspects of fNIRS (from hardware to analysis tools and paradigms) and the rise of new laboratories approaching fNIRS for the first time. Hence, more experienced fNIRS experts have the opportunity to advance the field by aiding trainees and providing critical feedback on the best ways to design the paradigm, process data (e.g., make suggestions about toolboxes or existing source codes), and reduce researcher degrees of freedom. The registered report format may also be of interest to graduate and early career trainees as it allows one to start the writing process and receive credit on one’s CV while initial lab setup, equipment orders, IRB applications, and paradigm development are underway. Furthermore, it provides protection to researchers and promotion of replications through the promise to publish accepted projects even if other similar research emerges in the period between paper acceptance and publication of results.

### 2.1 How Widely Adopted Is This Practice in the fNIRS Community?

To assess how many journals may be open to publishing neuroimaging registered reports, the Center for Open Science (COS) registered reports database^19^ was utilized. Here, out of the 261 journals listed on the COS website, 146 have previously published neuroimaging papers, and 44 have previously published fNIRS papers (see Supplemental Material for more details on our methods). Note, this is not the number of journals that have published registered reports, but rather journals that may accept neuroimaging registered reports for publication given their publication history. When searching for registered report papers that used fNIRS and have been accepted for publication, there were four research papers.^20^^–^^23^

### 2.2 Potential Challenges and Considerations

With the role of the reviewer taking on new responsibilities and contributing more feedback with this article format, it will be important to consider potential incentives or ways of recognizing the reviewer for their contributions (e.g., payment, badges, etc.). It is also critical for editors to identify reviewers who are knowledgeable enough in the particular subject area to make substantive suggestions and be able to mediate when reviewers (and possibly authors) make conflicting suggestions (e.g., different motion correction techniques, etc.).

### 2.3 Goals for the Future

Looking ahead, the fNIRS field could benefit from having this manuscript format more widely accepted by journals that typically publish fNIRS findings. In addition, journals can provide specific resources and checklists for reviewers to reference during each stage of review. Finally, the field may benefit from having a workshop at conferences, such as the Society for fNIRS (SfNIRS) meetings, or online webinars about the registered report format to further educate about and promote this practice.

## 3 Many Labs and Other Multisite Replicability Initiatives

The Many Labs and other multisite initiatives are a series of collaborative replication and methodological advancements projects (see Ref. 24 for an in-depth discussion on the goals and interpretation of replication studies). The first of these initiatives was the Open Science Collaboration (OSC, 2015) where teams of researchers across the globe attempted to replicate 100 studies from the field of psychology. They found that only 36% of the replications had significant results. Since then, several neuroimaging-specific replications and reproducibility studies have been conducted^25^^,^^26^ (see also Ref. 27) including a few ongoing efforts for fNIRS. The major theme coming out of the neuroimaging replication efforts is that even seemingly small decisions at various points in this process can substantively alter end results.^1^^,^^26^ In addition, these collaborative efforts have shed light on the importance of having adequately powered datasets.^28^ Overall, these results emphasize the need for more replication-based multisite initiatives alongside collaborative efforts to promote robust methods.

### 3.1 How Widely Adopted Is This Practice in the fNIRS Community?

Here, two current initiatives being made in the fNIRS community will be highlighted. The FRESH fNIRS Reproducibility Study Hub^29^ is a multilab initiative currently being launched by Robert Luke, Meryem Ayşe Yücel, and Rickson Mesquita. This project will provide participants with two fNIRS datasets and will ask participating teams to process and analyze the data as they see fit. There are currently representatives from over 100 institutions signed up to participate. The overall goal is to understand the variability in fNIRS processing and analysis strategies used across the field, and the potential consequences this has on data interpretation. Another initiative, Many Babies, is working to replicate developmental findings. One of these projects, ManyBabies 3 NIRS (MB3N), is a collaborative effort led by Judit Gervain to use fNIRS to understand the mechanism by which infants learn and apply rules (available in a GitHub repository at: https://manybabies.github.io/MB3N/). There are currently 30 active collaborators listed on the website for the MB3N fNIRS study and as a comparison, the related behavioral study, Many Babies 3, currently has 53 collaborators.

### 3.2 Potential Challenges and Considerations

Even with the growing institutional infrastructure, these multisite collaborative studies are very costly and time intensive. Therefore, careful cost-benefit analysis is needed when considering if a particular research question needs to be conducted on such a large scale or if the answers gathered in a single site study would suffice. Another potential challenge is authorship. With so many labs contributing to a particular project, it can be difficult to discern what the authorship order should be.

Another obstacle researchers face when running a replication study is differences in probe layout. Many researchers have 8 to 16 sources and 8 to 16 detector systems that only allow coverage for two to three regions of interest. Given the sparsity of channel coverage, it can be difficult to cover all regions necessary to run the replication study. In addition, different replications focusing on different regions of interest could require additional purchases (e.g., new caps with coverage for the different regions of interest). Slight variations in cap layout (e.g., differences in source and detector layouts, cap placements, and channel distances) can also lead to the failure to replicate so careful considerations for cross-site replications would need to be made.

### 3.3 Goals for the Future

Looking ahead, it will be exciting to see the results of the current fNIRS Many Labs’ initiatives. Beyond identifying which studies replicate, it will be informative to investigate reasons as to why particular results do or do not replicate. A “failed” replication might provide a reason to doubt the phenomenon described in the original study, but because no two studies are exactly alike, the replication efforts may also uncover new information about the differences that lead to divergent results. Divergent findings between the original and replication study could point to many interpretations beyond the original study being “wrong” and the new replication study being “right.” Nonetheless, both studies could be scientifically sound but differ in some aspects that were previously unknown, overlooked, or that have changed over time (e.g., participant demographics, cultural context, cohort effects, differences between NIRS acquisition systems). Alternatively, one or both studies could present flaws in their methodology (e.g., under powered studies, flaws in the design or analysis^24^). Notably, fNIRS research constitutes a decision tree, from the selection of acquisition hardware and stimuli to analytical choices in data cleaning and processing, and any number of these seemingly small decisions could plausibly contribute to variation in outcomes between studies. Therefore, analyzing the factors leading to divergent results, provides an opportunity to strengthen research practices in all fields, NIRS included (for more information on replication studies see Ref. 24).

In addition, the hope is that these collaborative efforts will bring the fNIRS community closer together, get more fNIRS researchers involved, and create the necessary infrastructure to support future cross-site initiatives. As the field grows, there are a larger number of experts and sites with the necessary resources and equipment to conduct large, multisite studies. Hence, fNIRS could be integrated into other large-scale studies such as Adolescent Brain Cognitive Development (ABCD^30^) and Healthy Brain Child Development,^31^ which have thus far focused on EEG and fMRI data collection due in part to these methodologies having a wider user base. For this, it would also be of help to have more cross-lab standardization of cap layouts that are available at an affordable cost.

## 4 Sharing Materials

Sharing materials can take many forms including sharing stimuli, paradigm presentation scripts, preprocessing streams, and analysis code. In the 30 years of fNIRS research, paradigms, preprocessing streams, and analyses have become increasingly complicated and reliant on in-depth programming knowledge.^32^ Requiring such a knowledge base creates barriers to entry for new researchers and is even prohibitive to more experienced researchers who relied upon more traditional methods. Therefore, it would be helpful for the field to have methods widely disseminated and in a user-friendly (step-by-step) manner (see Ref. 33 for tips on code sharing). In addition, sharing materials promotes replicability and aids advanced researchers to refine or build new methods inspired by others in the fNIRS community. Sharing such tools and materials (see Refs. 34 and 35, for examples, of sharing fNIRS presentation paradigms) has become easier across all fields with code sharing databases such as GitHub, and fNIRS-specific code sharing databases (e.g., Ref. 36). In addition, *Neurophotonics*, the official journal for the Society for fNIRS, already promotes code sharing through its acceptance of tutorial manuscripts and also provides suggestions for code sharing on its website (see https://codeocean.com/signup/spie).

### 4.1 How Widely Adopted Is This Practice in the fNIRS Community?

**Fig. 2:**
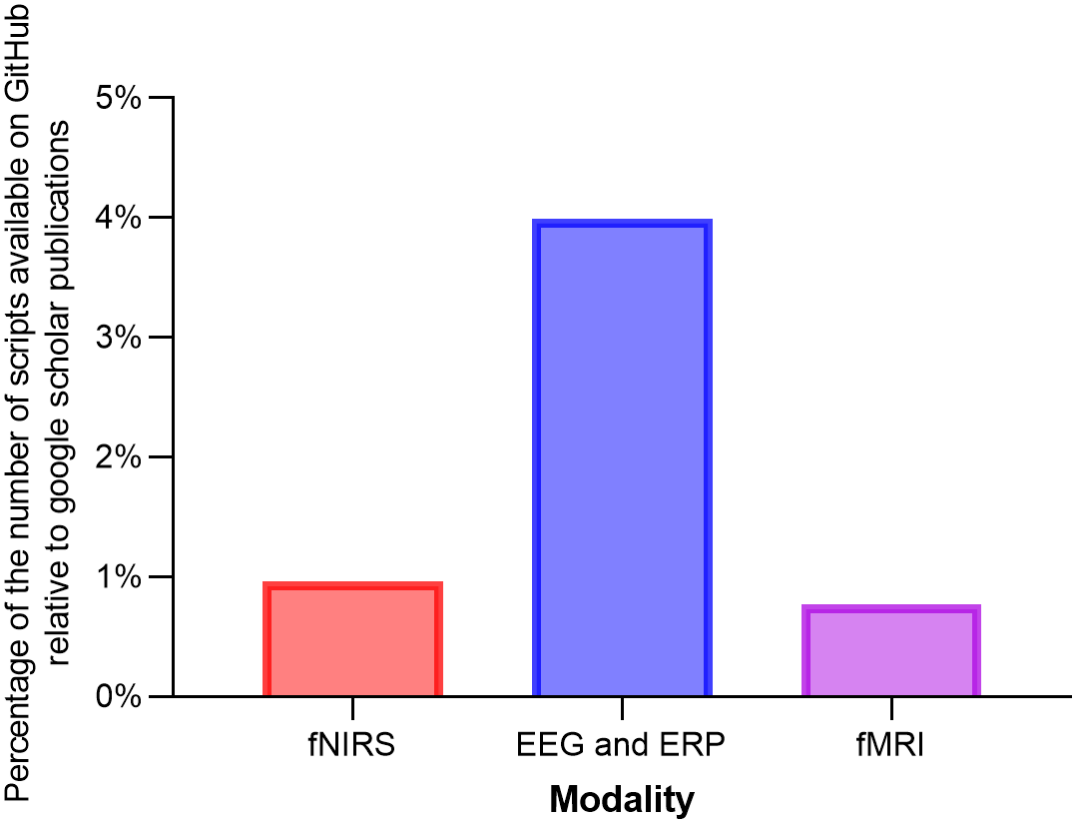
The ratio of codes available on GitHub relative to publications for each imaging modality. Note. This search was conducted on March 29, 2022, using GitHub. The following search terms were used: fNIRS *or* “functional near-infrared spectroscopy,” fMRI *or* “functional magnetic resonance imaging,” EEG *or* electroencephalogram *or* ERP *or* “event related potential.”

Overall, there were substantially fewer repositories shared on GitHub about fNIRS compared with other modalities (see Table S2 in the Supplemental Material). However, when accounting for the total number of Google Scholar publications (used as an approximation for users across these modalities; see Fig. 2), fNIRS and fMRI show similar rates of code sharing. In terms of the types of software being shared, the majority of code in Ref. 37 is made primarily for fNIRS data processing (n=16), but there is also software available to aid in probe development (n=3) and specialized analyses (e.g., functional connectivity; n=1).

### 4.2 Potential Challenges and Considerations

There are potential negative consequences for widespread material sharing, including a risk of disproportionality benefitting well-resourced labs. For example, smaller labs that possess the skills to develop and share processing scripts may not be able to benefit from the analysis pipeline as quickly as other labs with greater funding and staffing. Standardization of stimuli could also lead to problems with generalizability. For example, if all labs adopted the Nim Stim set of facial expressions^38^ to study neural responses to emotional faces, it is possible that the brain responses identified as being relevant to fearful faces are actually in response to a specific element of the stimuli (e.g., hair is or is not present in the pictures, luminance features, etc.). In addition, not all stimuli can be readily adopted for all studies (e.g., they are not culturally relevant).

### 4.3 Goals for the Future

The field can work to have an agreed-upon format for code sharing. Journals may even want to go one step further by expecting code sharing but allowing for reasonable exceptions. Moreover, fNIRS researchers can continue to strive to make fNIRS research more accessible through the use of freely available software (e.g., using Psychopy for stimulus presentations, developing programs, such as HOMER—that can be used on MATLAB Runtime without requiring a license, creating fNIRS processing packages for R or Python, etc.). In addition, more support for programming and materials sharing can be provided through hosting educational sessions (such as HOMER3 tutorials and sfNIRS educational events at conference meetings) and creating coding curricula for undergraduate and graduate studies.

Another option for sharing protocols is through sharing videotapes of testing sessions. This may allow secondary data users insights into slight nuances of the paradigms and testing conditions that are difficult to glean from testing manuals.^39^ One resource available to support these efforts is Databrary.^40^ Databrary is a cloud storage system that assists with the storage, streaming, and sharing of audio and visual recordings collected as research data or documentation. This resource could be of interest to fNIRS researchers because it would allow researchers to upload videos of the fNIRS setup, cap placement, and the testing session to document these protocols for their own lab and to share with other researchers interested in replicating the protocols (although see the potential challenges and considerations section of data sharing for a discussion on the challenges of sharing identifiable data). Sharing of materials can also be encouraged by sponsoring calls for papers with tutorials focused on advanced fNIRS processing and methodologies that solve common issues faced by specific fields (see Ref. 41 for an example).

Finally, safeguard and rewards systems can be put in place so one type of lab is not disproportionally benefitting from the practice. One example safeguard is allowing for embargo periods. That way research groups can release the codes once they have had the time to run the original analyses that they planned. Another way to help prevent possible inequities caused by sharing materials is through developing an incentive structure such as badges and having them be a criterion for reviewers to assess when making suggestions for publication. Incentives could also be promoted at the institutional level; for example, hiring and promotion committees could reward stimuli and software creation (e.g., creating a highly cited software package could be seen as the equivalent of a highly cited paper).

## 5 Sharing Data

Data sharing refers to posting raw fNIRS data to publicly accessible repositories, and this practice is of great importance because it facilitates secondary data analysis and meta-analytic efforts. Moreover, data sharing can help to improve the reproducibility of research by allowing others to identify mistakes and provide suggestions for improved analyses. These efforts can also support student or early career researchers that may not have the resources to collect their own data but have an interesting theoretical question that can be asked using someone else’s data. There are a variety of online repositories that support the storing and sharing of functional neuroimaging data. (These are a few data repositories: Data Archiving and Networked Services, Dataverse, fighshare, NeuroImaging Tools & Resource Collaboratory, OpenNeuro, the Open Science Foundation, Zenodo, among others.) Because the structure and format of fNIRS data can vary across acquisition systems, processing streams, etc., the Society for fNIRS has proposed the Shared Near InfraRed File (SNIRF) as the official file format for fNIRS data (Ref. 42 and available in a GitHub repository at: https://github.com/fNIRS/snirf/). SNIRF is a standard, universal, HDF5 format supported by a variety of common languages and programs (MATLAB, including HOMER3; Python, including MNE, etc.). An increasing number of fNIRS devices currently on the market will automatically output data into the SNIRF format and scripts are available to convert older file formats (e.g., .nirs) to the SNIRF format. For example, Homer3 will automatically load and convert .nirs files upon the start-up of the program. Another effort at standardization involves how data are organized and a current push is being made to create a Brain Imaging Data Structure (BIDS) extension specific to fNIRS data (and utilizing the SNIRF format), which as of publication is nearing its final stages of review and release (available in a GitHub repository at: https://github.com/bids-standard/bids-specification/pull/802). The creation of the BIDS format will be of great use, as platforms such as OpenNeuro require data to be BIDS-compliant.

### 5.1 How Widely Adopted Is This Practice in the fNIRS Community?

Currently, there are 12 (the ratio of codes shared to Google Scholar publications = 0.00042) data files available on the openfNIRS website^43^ and seven (the ratio of codes shared to Google Scholar publications = 0.00024) available in Ref. 44. However, there are a larger number of datasets for other neuroimaging modalities. Although the numbers are relatively similar when considering the dataset per user ratio (when accounting for usership with Google Scholar publications). Specifically, there are 488 fMRI (the ratio of codes shared to Google Scholar publications = 0.00110) and 81 EEG (the ratio of codes shared to Google Scholar publications = 0.00008) datasets available on Open Neuro,^45^ alone (see Ref. 46 for an examination of the frequency of data sharing across fields). For the currently available data on openfNIRS, the majority are saved in the .snirf format (n=10 snirf, n=1 csv, n=1 not listed), and the more recently posted data follow the BIDS organization (n=3).

### 5.2 Potential Challenges and Considerations

Across data platforms, there are debates as to if data should be shared in the raw or processed (e.g., concentration change values for each condition already computed). There are pros and cons to either storage method. Data stored in the raw format allow other researchers to perform their own processing stream and permit greater analytical flexibility. Processed data, on the other hand, reduce the barrier to entry for secondary data analysis because they do not require advanced knowledge of fNIRS signal processing. It could also ensure greater consistency across secondary analyses of the same underlying data. Unlike the SNIRF format for raw data, there are no standardized formats or structures for sharing processed data. A standardized template for processed data might include variable labels, data type (e.g., whether data reflect individual trials or averages across trials, whether time series data are available for waveform reconstruction), chromophore inclusion (i.e., whether data reflect values from oxygenated and/or deoxygenated hemoglobin), channel location information (e.g., whether data reflect individual channels or regional averages, whether probe layouts are provided), and various metadata (e.g., head circumference, acquisition hardware/software).

There are also important ethical and financial considerations that can arise when sharing data. For example, some countries, institutions, and funders have stricter data sharing policies than others, so sharing data is not always possible or requires additional infrastructure (e.g., legal resources). Another consideration is that researchers must ensure that participants are fully informed about the scope and specificity of the data that will be shared, as well as the public nature of online repositories (see Refs. 47 and 48 for templates of consent form language for sharing data with other researchers). Users of secondary data must also be careful when handling data, especially if those data are identifiable or potentially identifiable (e.g., photos, zip codes, job descriptions).^49^ Asking participants to share identifiable data (zip code, photos, job description, birthday, etc.) could also create a risk for sampling biases if, for example, people from historically disadvantaged groups feel less comfortable sharing identifiable data.^50^ Thorough and individualized approaches to the informed consent process can ensure that participants are able to make fully informed decisions about their participation and data.^50^

Another consideration with posting data and including it as part of a paper’s publications is clarifying if and how this should be examined during the peer review process (e.g., if reviewers are responsible for reviewing the data files). A related issue, if data files are part of the reviewing data files and rerunning statistical analyses does become a routine task for reviewers, there will need to be a consideration for how to recognize and reward this increased workload. If there is an increase in demand but not an increase in support for peer reviewers, then it is possible that significantly fewer reviewers will volunteer, and the quality of peer review will decrease.

### 5.3 Goals for the Future

In the future, formal guidelines for sharing data can be created. By creating a uniform structure, researchers may be able to more easily merge datasets that are differing in acquisition systems and probe layouts. In addition, data sharing can be incentivized by recognizing researchers who have posted their data through a badge program, and by making data availability a criterion upon which reviewers assess a manuscript (see also this editorial on recognizing authors of public datasets, “Time to recognize authorship of open data”). In addition, data sharing platforms can continue to strive to become increasingly accessible and streamlined into the everyday research cycle so there is not too large of a time or financial (e.g., requiring a full-time data manager) burden on the researcher. Importantly, platforms can be designed in such a way that they are well suited for all stages of one’s career, such that each platform is easy to use regardless of the dataset size (e.g., a single condition fNIRS study with five participants or a multisite collaboration with thousands of participants). Furthermore, it is recommended to develop educational resources for researchers (e.g., templates that use easy-to-understand and thoughtful language for consent forms for the release of fNIRS data), host outreach programs to help educate the public on the costs and benefits of data sharing, and host sessions at sfNIRS where panelists discuss ways in which researchers have been successful (or not so successful) in navigating issues related to ethics, data ownership, and data sharing.

Moving forward, it will also be helpful to expand resources for de-identifying information that is critical to analyzing fNIRS data. For example, participant-level data on probe placement is critical to ensuring that data reflect the intended scalp/brain regions. However, because cap placement is often documented through photographs or video recordings, these data are often unable to be shared. Therefore, it would be of great help to the community to continue to create easy-to-implement and user-friendly programs capable of recording and sharing nonidentifiable cap placement information. One example of this is STORM-Net, which has been developed to convert spatial information from video recordings into unidentifiable data files of MNI coordinates.^51^

## 6 Preprints and Open Access

A preprint is a complete manuscript posted to a publicly accessible web service without having undergone peer review. Many manuscripts posted as preprints are also submitted for subsequent publication as traditional journal articles. Preprints allow for findings to be shared expediently and without a cost to the reader.^52^ Preprint services also help address the file drawer problem^53^ because they allow for dissemination of papers with nonsignificant findings. As part of a related initiative, some journals allow articles to be published as open access (i.e., accessible to the public without a fee/subscription). *Neurophotonics*, the official journal for the Society for fNIRS, has made all articles open access.

### 6.1 How Widely Adopted Is This Practice in the fNIRS Community?

**Fig. 3:**
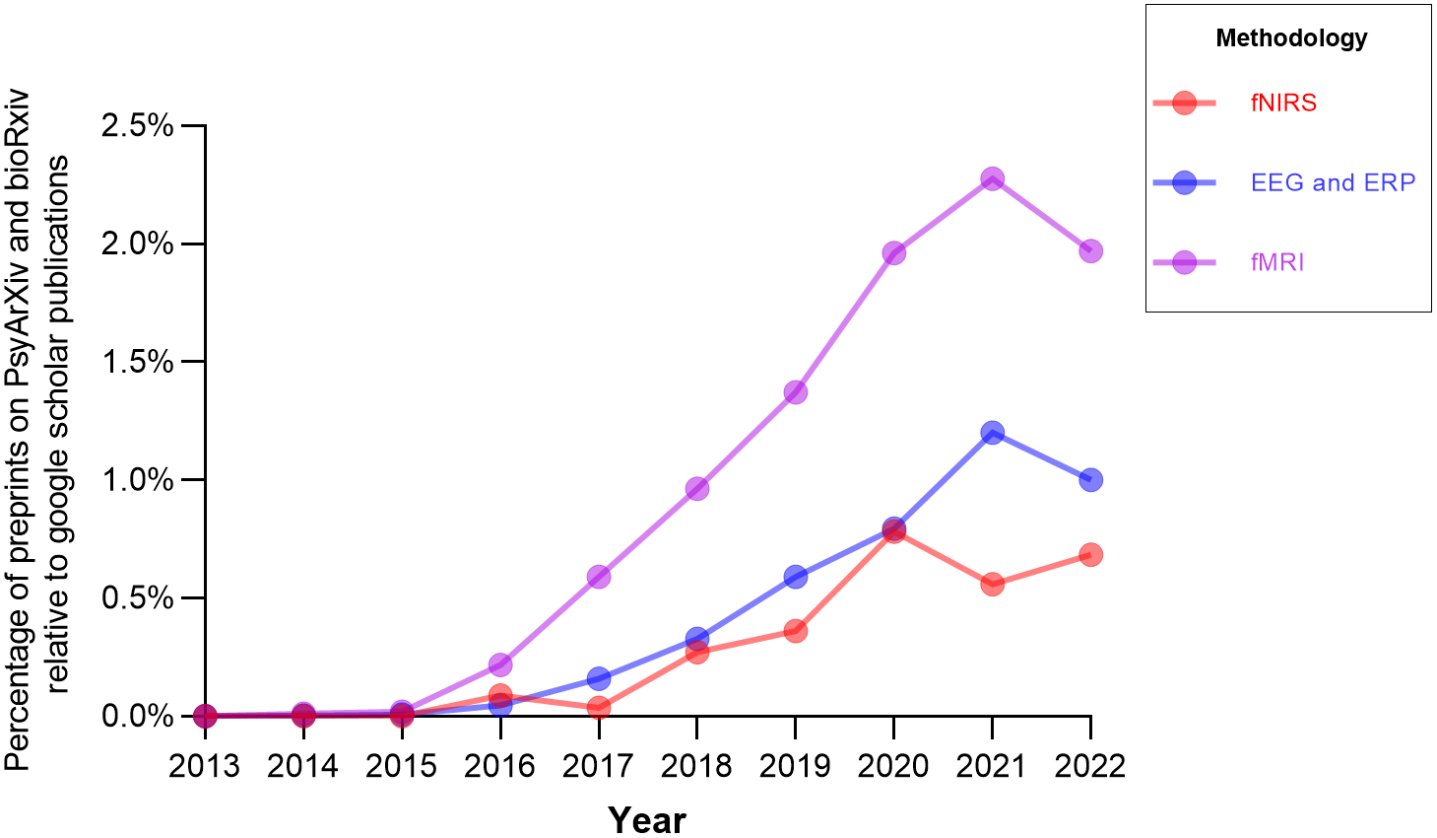
The ratio of preprints to publications for each imaging modality by year. Note. This search was conducted on March 29, 2022, using Ref. 10 (for PsyArXiv) and bioRxiv; please see the Supplemental Material for more details on these searches. The following search terms were used for PsyArXiv: fNIRS *or* “functional near infrared spectroscopy,” fMRI *or* “functional magnetic resonance imaging,” EEG *or* electroencephalogram *or* ERP *or* “event related potential.” The following search terms were used for bioRxiv: fNIRS, fMRI, EEG, *or* ERP.

To quantify how fNIRS is doing relative to other methodologies a search was performed using two popular preprint sites, *bioRxiv* and *PsyArXiv*. Overall, there were significantly fewer preprints written about fNIRS (see Table S3 in the Supplemental Material) compared with other methodologies, and this pattern held even when accounting for the total number of publications on Google Scholar (used as an approximation for users; see Fig. 3). In addition, there was an upward trend such that preprints are becoming generally more common across all three imaging modalities.

### 6.2 Potential Challenges and Considerations

It is important to consider some potential problems with preprints; for example, articles published as preprints are not peer-reviewed and could therefore be more prone to methodological problems and analytical errors (but see Refs. 54 and 55 for discussions of potential problems with current peer review practices and of other ways to prevent errors in scientific publication). This may also lead to findings being disseminated and interpreted by general public prematurely. Another potential downside is that preprints contain identifying information that can bias the peer review process.

There are also important economic and ethical concerns related to open access publishing. For example, the financial burden of open access publication often falls on the authors of the papers. The price varies across journals, but it can range from hundreds to thousands of dollars, and this cost can be prohibitive for many researchers. Moreover, there are concerns about the fairness and equity of authors having to secure their own research funding, to pay for findings to be published, and to volunteer as peer reviewers, while not receiving compensation from journals for their publications and reviews.

### 6.3 Goals for the Future

In the future, it will be important to educate the public and the media on the potential for error that comes with articles not going through the peer review process. To this end, journalists could be encouraged to state in their reporting that “the findings have not yet been peer-reviewed” prior to describing the results of the study. The field could also consider creating a blinding system for preprints to keep the author’s identity anonymous while undergoing the peer review process and establishing more funding mechanisms to support publishing in open access journals.

## 7 General Conclusions and Suggestions for the Future

In this review, the important advances the fNIRS community has made in participating in the various initiatives of open science are highlighted. Although open science practices in the fNIRS field have become more common in recent years, there is ample room for wider adoption. To advance the fNIRS community’s participation in open science, the authors propose a number of general suggestions for the future. First, the authors propose the development of a working group for open science in fNIRS, in which researchers gather periodically to review the status of the field’s practices and compile recommendations and resources to help other researchers more easily participate in open science. Second, the authors recommend that open science education sessions are offered regularly, possibly at sfNIRS meetings and online webinars, to make researchers aware of best practices and provide resources to support the adoption of these practices. Third, the authors propose that open science practices can be specifically highlighted in job advertisements and valued in faculty searches to provide incentives for researchers to participate in open science. A related suggestion is that researchers may consider highlighting their open science practices on their CVs and on their lab websites by indicating how they have promoted open access by sharing code and stimuli (e.g., sharing links to their OSF or GitHub page) or have participated in transparent practices such as writing preregistrations and registered reports (e.g., putting an extra star next to publications where the methods and analyses were preregistered). Fourth, the authors propose contributing financial (e.g., writing into grant budget, soliciting donations) resources to support open science platforms (e.g., preregistration, preprint services, and data repositories). If these efforts are not supported, then there is the possibility the platforms will no longer be able to provide their specific services.

By adopting these suggestions, the fNIRS community can take steps toward advancing transparency, reproducibility, rigor, and efficiency in the field. Overall, this review hopes to demystify various stages of open science and clarify the current state of open science in the fNIRS field.
